# Clip-to-clip hand-in-hand closure method for a large mucosal defect after colorectal endoscopic submucosal dissection

**DOI:** 10.1055/a-2409-5004

**Published:** 2024-09-25

**Authors:** Xiaoyan Lv, Guodong Li, Hongbo Ren

**Affiliations:** 1 66310 Department of Gastroenterology, The First Affiliated Hospital of Shandong First Medical University, Jinan, China

Complete closure protects patients with large non-pedunculated colorectal lesions with a high risk of bleeding and coagulation syndrome after endoscopic submucosal dissection (ESD). The rectal mucosa and muscle layers are relatively thick, making it challenging to completely close large rectal wounds with conventional clips 1 . This report describes a novel clip-to-clip hand-in-hand closure method ( Fig. 1 , Video 1 ).

**Fig. 1:**
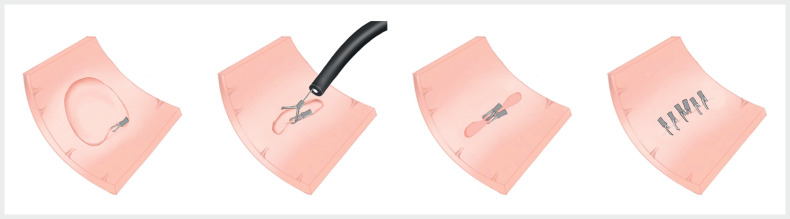
Schematic diagram of the clip-to-clip hand-in-hand method assisting with large wound closure after endoscopic submucosal dissection (ESD).

Clip-to-clip hand-in-hand closure method facilitates complete closure of a large defect after colorectal endoscopic submucosal dissection.Video 1

An 87-year-old woman with abdominal distension underwent a colonoscopy, revealing a 40-mm laterally spreading tumor in the rectum. After excluding contraindications, ESD was performed successfully ( Fig. 2 ). Due to the patient’s advanced age, hypertension, long-term antiplatelet therapy, and large lesion size, she was identified as a high risk for post-procedural bleeding 2 . To mitigate this risk, a complete prophylactic wound closure was undertaken. Initially, a rotatable clip (Hemoclip, 11-mm opening; Vedkang Medical, Jiangsu, China) was applied to grasp and close the anal-side mucosa and submucosa ( Fig. 3 **a** ). The second clip was opened, and one prong was inserted into the gap of the first closed clip, making it an “anchor.” By pulling the second clip, the other prong of the second clip grasped the opposite mucosa, assisted by suction ( Fig. 3 **b** ). After closing the second clip, the wound was narrowed ( Fig. 3 **c** ). For larger wounds, this process was repeated to transform the round wound into a strip shape ( Fig. 3 **d** ), using additional clips for complete closure ( Fig. 3 **e** ). The patient was discharged on the 5th postoperative day with no abnormalities at the 2-week follow-up.

**Fig. 2:**
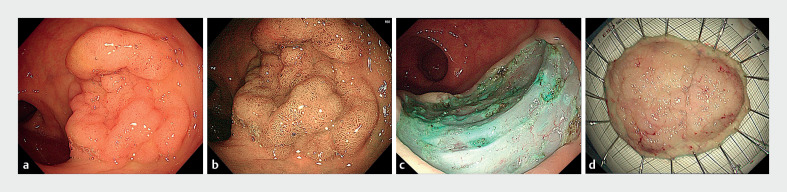
Images of lesion and ESD procedure. **a** A 40-mm laterally spreading tumor in the rectum. **b** Narrow-band imaging view. **c** Wound after endoscopic coagulation. **d** A 50 × 46-mm specimen was resected by ESD.

**Fig. 3:**
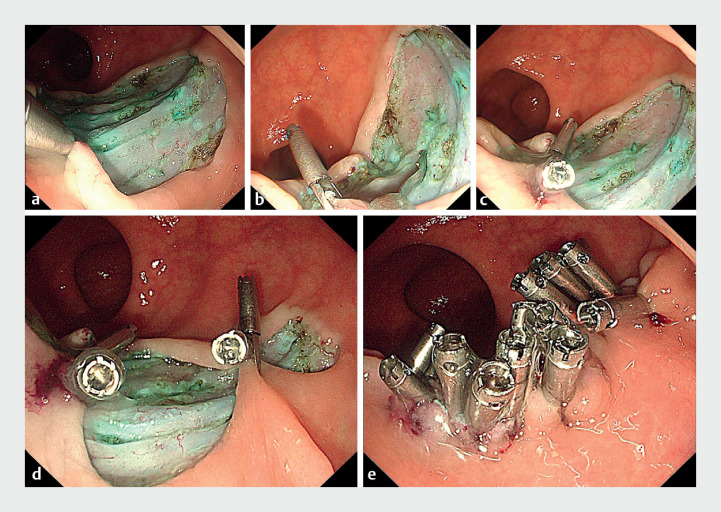
Images of the clip-to-clip hand-in-hand closure procedure. **a** A clip grasped some anal-side mucosa and closed it. **b** One prong of the second clip passed through the gap of the first closed clip; by pulling the second clip, the other prong grasped the opposite mucosa. **c** After closing the second clip, the wound was narrowed. **d** Above process was repeated to transform the round wound into a strip shape. **e** Complete closure was easily achieved with additional clips.

The clip-to-clip hand-in-hand closure method simplifies and enhances the clip-on-clip closure technique 3 . It functions similarly to a “dual-action clip,” being simple and efficient and not requiring high technical skills or endoscope withdrawal for additional tools. This method is suitable for closing large wounds with high bleeding risks throughout the gastrointestinal tract and offers economic advantages.

Endoscopy_UCTN_Code_TTT_1AQ_2AG

## Notes

Endoscopy E-Videos https://eref.thieme.de/e-videos
*E-Videos* is an open access online section of the journal *Endoscopy* , reporting on interesting cases and new techniques in gastroenterological endoscopy. All papers include a high-quality video and are published with a Creative Commons CC-BY license. Endoscopy E-Videos qualify for HINARI discounts and waivers and eligibility is automatically checked during the submission process. We grant 100% waivers to articles whose corresponding authors are based in Group A countries and 50% waivers to those who are based in Group B countries as classified by Research4Life (see: https://www.research4life.org/access/eligibility/ ). This section has its own submission website at https://mc.manuscriptcentral.com/e-videos .
